# PBF/PTTG1IP coordinates focal adhesion formation, polarity and cell motility

**DOI:** 10.64898/2026.09.14.751110

**Published:** 2026-09-16

**Authors:** Merve Kocbiyik, Selvambigai Manivannan, Davina Banga, Aditi Hariharan, Mohammed E El-Asrag, Andrew D Beggs, Paloma Garcia, Dean P Larner, Saroop Raja, Jeremy A Pike, Afshan Afzal, Katie Brookes, Hannah R Nieto, Steven G Thomas, Martin L Read, Christopher J McCabe, Vicki E Smith

## Abstract

Directional cell migration requires cells to sense extracellular cues and coordinate adhesion, cytoskeletal remodelling and polarity, yet the molecular regulators that integrate these events remain incompletely defined. PTTG1-binding factor (PBF/PTTG1IP) is a transmembrane glycoprotein extensively characterised in pathological overexpression and cancer models, where elevated expression promotes tumourigenic cellular phenotypes. However, its endogenous physiological function remains poorly understood. Here, separate enrichment analyses of transcriptomic and phosphoproteomic profiles from PBF-overexpressing cells collectively highlighted cell adhesion, extracellular matrix organisation, cytoskeletal regulation and cell motility as major PBF-associated programmes. Using a novel Pbf knockout mouse model, we show that primary Pbf-KO mouse embryonic fibroblasts exhibit impaired migration and invasion. PBF loss reduced fibronectin adhesion, disrupted focal adhesion number, size and distribution, attenuated FAK Tyr397 phosphorylation and delayed early adhesion formation. Live-cell imaging and Golgi orientation assays further revealed altered actin organisation and impaired front–rear polarity in Pbf-KO cells. Key phenotypes were conserved in CRISPR–Cas9 PBF-KO human thyroid cancer cells, while targeted PBF re-expression restored migration in Pbf-KO MEFs. We thus identify PBF as an endogenous regulator of cell adhesion, polarity and directional motility, providing a framework for understanding how pathological PBF overexpression may promote invasive behaviour in cancer.

## Full Text Availability

The license terms selected by the author(s) for this preprint version do not permit archiving in PMC. The full text is available from the preprint server.

